# Cultural differences in the use of acoustic cues for musical emotion experience

**DOI:** 10.1371/journal.pone.0222380

**Published:** 2019-09-13

**Authors:** Vishal Midya, Jeffrey Valla, Hymavathy Balasubramanian, Avantika Mathur, Nandini Chatterjee Singh

**Affiliations:** 1 Language, Literacy, and Music Laboratory, National Brain Research Centre, Manesar, Haryana, India; 2 Division of Biostatistics and Bioinformatics, Department of Public Health, Penn State College of Medicine, Pennsylvania State University, Hershey, Pennsylvania, United States of America; University of Wroclaw, POLAND

## Abstract

Does music penetrate cultural differences with its ability to evoke emotion? The *ragas* of Hindustani music are specific sequences of notes that elicit various emotions: happy, romantic, devotion, calm, angry, longing, tension and sad. They can be presented in two modes, *alaap* and *gat*, which differ in rhythm, but match in tonality. Participants from Indian and Non-Indian cultures (N = 144 and 112, respectively) rated twenty-four pieces of Hindustani *ragas* on eight dimensions of emotion, in a free response task. Of the 192 between-group comparisons, ratings differed in only 9% of the instances, showing universality across multiple musical emotions. Robust regression analyses and machine learning methods revealed tonality best explained emotion ratings for Indian participants whereas rhythm was the primary predictor in Non-Indian listeners. Our results provide compelling evidence for universality in emotions in the auditory domain in the realm of musical emotion, driven by distinct acoustic features that depend on listeners’ cultural backgrounds.

## Introduction

Music is “humanly organized sound”, a means of communicating emotions and present in some form in every known human culture [1,2]. Indeed, descriptions of music as a universal ‘language’ of emotions have garnered empirical support [3–6] and thereby offer a unique format to explore universality in emotion in the auditory domain. Past studies of music and emotion across cultures [7] have posited an interplay between two primary factors in music emotion recognition (1) familiarity with the conventions of a culture’s tonal system, and (2) psychophysical acoustic cues which communicate emotion irrespective of cultural experience. According to the cue redundancy model (CRM) [7] native and non-native listeners can decode the psychophysical acoustic elements conveying emotion in a piece of music, but the expression of emotion in a given piece is most salient to listeners of that same culture, who have the added benefit of familiarity with culturally-related acoustic cues which reinforce, redundantly, the psychophysical acoustic cues.

Two acoustic universals which are commonly cited as putative universals that define music and carry both cultural and universal emotional cues are tonality and rhythm [8,9]. Tonality refers to the pitch frequencies of the notes (in Hindustani/North Indian classical music, *swaras*) in a piece of music, and the relationships between them. For instance, the associations between (in Western terminology) major intervals and positive or excited emotion, and minor intervals and negative or subdued emotion, are consistent in Western music [10,11] and Hindustani classical music [12,13]. Rhythm includes the spacing of notes across a piece of music in a regular and repeated pattern. The ability to entrain movement to musical rhythms and tempos is universal even though the structure of musical rhythms differs between cultures as illustrated by past studies which have shown that faster fluctuations in rhythm and faster tempo convey happiness, while the slower ones convey sadness [14,15].

Despite these specific roles played by tonality and rhythm, even the casual music listener can conclude that the emotions elicited by music cannot be restricted to singular domains of tonality and rhythm, but rather the interplay between them. However, the degree to which these two factors influence the experience of musical emotions, and whether the relative influence of one factor versus the other differs between culturally familiar and unfamiliar listeners, is unclear.

Hindustani music is a musical system comprising a canon of *ragas*, which are specific sequences (melodies) of *swaras* (notes) (for a detailed review see [16]). These *ragas* are presented in two successive stages: the *alaap*, followed by the *gat*. The *alaap* slowly introduces the *swaras* of a *raga*, bound strictly to the defining sequence of the *raga*, but not bound by any prescribed rhythm; performers are free to improvise accentuations and spacing of the *swaras*, as long as they stay within the boundaries of the *raga* sequence. The *alaap* is rendered on a melody instrument or vocally, backed by an accompanying drone which supplies the tonic “root” note. Following the conclusion of an *alaap*, the *raga* transitions to the *gat* stage, which uses the same melodic sequence of *swaras*, but rendered at a faster tempo and with the accompaniment of a percussion instrument which bounds the piece to a stricter, explicit rhythmic cycle [16]. In this way, the structure of Hindustani *ragas* are uniquely structured to tease apart the emotional influences of tonality and rhythm [17]. This is because a *raga* is an inherently experimental stimulus: it is performed in two successive rhythmic “stages” (*alaap* and *gat*), while the tonality of the piece is controlled for across these “conditions”. (S1 Appendix, S1 Table)

Past cross-cultural studies of emotional responses to *ragas* have indicated that even participants from other cultures accurately perceive the intended emotions of joy, sadness and anger in *ragas* [18,19]. Specifically, Laukka et al., [19] showed that not only Indian, but also Japanese and Swedish participants were able to recognize the intended emotions of different *ragas* at above chance levels. Specifically, Japanese listeners were adept at identifying anger and humor, whilst Swedish listeners recognized anger, humor, longing, peacefulness and sadness. All studies conducted with non-enculturated participants so far, used only the *alaap*, namely the introductory arrhythmic section of the *ragas*. Recent research by Mathur et al., [13] has shown that *alaap* elicits limited emotional responses while the *gat* produces a wide range of emotional responses. In a free response rating task, they found that while *alaap* pieces were rated calm, sadness and happy, *gat* elicited additional emotional responses like longing, tension, romance and devotion in addition to calm, sadness and happy. Since past studies have not included *gat*, a cross cultural comparison of emotional responses elicited by *alaap* and *gat* allows an investigation of emotions across different stages of the same *raga*.

A secondary important question of interest in the domain of cross-cultural emotion research is to compare the usage of emotion labels to describe the emotional response. While Fritz et al., [6] showed universals in the recognition of basic emotions like happy, sadness and fear, Scherer and Zentner [3] have remarked that emotions experienced in response to music differ from basic emotions. They speculate that emotions arising from music are more appropriately categorised as ‘aesthetic’, resulting from an appreciation of the intrinsic qualities of music [20]. Since cultures differ in terms of the levels of emotional spontaneity and expressive display that are considered appropriate [21], important cross-cultural differences may arise in the experience and use of emotion labels used to describe the emotions aroused by music. For instance, a study by Benamou [22] comparing terminologies used to describe Javanese music with that used by Hevner’s adjective circle which used terms from European music revealed both similarities in basic categories, and interesting differences between cultures. Hevner's ‘sad’ cluster consisted of the following adjectives: dark, depressing, doleful, frustrated, gloomy, heavy, melancholy, mournful, pathetic, and tragic [23]. In Javanese music, on the other hand, sadness translated as ‘sedhih’ was associated with the words: plain, calm, devotional, moving, full of pity, pained, grief-struck, despondent, bewildered, troubled, in love, yearning, lonely, desolate, and chilly. On comparing the two terminologies, Benamou [22] explained that the terms used in Hevner’s ‘sad’ cluster, (e.g. ‘dark’ or ‘tragic’) were not common Javanese adjectives for sadness. Similarly, ‘tragic’ was not a common musical or dramatic mood aroused by Javanese music. This suggests that, while terms used to describe aesthetic emotions linked to music may differ across cultures [24], this phenomenon has been little investigated. Consequently, one of the objectives of this study was also to investigate the use of emotional labels across cultures while labelling experienced emotional response.

As described above and demonstrated earlier [13], the *ragas* of Hindustani music are not only uniquely structured to investigate the effect of rhythm and tonality on emotional response but since they elicit a gamut of emotional response, they also allow an extensive comparison of musical emotion labels across cultures, a previously unexplored venture.

To summarize, the two major objectives of this study were (1) To compare both labels and intensity of emotional responses in two presentation phases of *raga* music (*alaap* and *gat*) between culturally familiar, and unfamiliar listeners—hereafter referred to as ‘enculturated’ and ‘non-enculturated’ listeners. (2) To delineate the relative roles of tonality and rhythm in determining emotional responses in both groups of listeners. In accordance with earlier studies [6,25] and the CRM model [7], we hypothesized similar emotional responses by non-enculturated and enculturated participants for both *alaap* and *gat*. We also speculated that the most culturally relevant acoustic cue to emotion would be evidenced in the greatest differences between enculturated and non-enculturated groups in terms of whether tonality or rhythm explained greater percentages of emotion ratings.

## Materials and methods

### Online music emotion survey

With the purpose of collecting data from all around the world, a survey consisting of three-minute instrumental renditions of twelve *ragas* was conducted online. These *ragas*, played on a stringed instrument (*sarod*), were digitally recorded in both the *alaap* and *gat* stages. The task of the participants was to listen to the *raga* excerpts and rate their emotional experience on Likert scales of 0–4 (0 being ‘not at all felt’ to 4 being ‘felt the most’ for eight emotion labels: happy, romantic, devotional, calm/soothed, angry, longing/yearning, tensed/restless, and sad. The study was approved by the Institutional Ethics Committee of the National Brain Research Centre.

The *ragas* selected for the study were played by a professional musician on *sarod* (a stringed instrument) digitally recorded in *alaap* and *gat*. The *sarod* was tuned by the artist to just intonation scale with the tonic being at 261.5 Hz. The emotion labels used in the study were translated to Hindi and transliterated to Roman. The emotion labels used along with the respective translation to Hindi and transliteration to Roman are as follows: happy (*khush*), romantic (*premmaye*), devotional (*bhaktipurn*), calm/soothed (*shant*), angry (*krodhit*), longing/yearning (*virahapurn*), tensed/restless (*bechain*), and sad (*udas*). The Romanized transliteration was used as it is widely used in internet communication and global commerce [26].

### Experimental design

The *raga* excerpts heard by participants of the online survey were presented in alternating *alaap* and *gat* blocks. The experiment consisted of four such blocks with each block consisting of six *ragas*. The presentation of *alaap* or *gat* block as the first block was counterbalanced across subjects. The order of presentation of *ragas* within each block was randomized across participants. The participants were given an option to opt out of the survey after rating at least two blocks (i.e., 12 *ragas*–six *alaap* and six *gat*). The survey was made available at https://nandinisingh.wixsite.com/labweb/musicemotion.

### Participant demographics

650 participants registered for the survey over the period of two years; however, only data from participants who completed at least half the survey [i.e., rated at least six *alaap* excerpts (out of 12) and six *gat* excerpts (out of 12)] was considered for analysis. Thus, the analysis reported in this study is based on the emotional responses reported by 255 participants (118 males, 137 females), 143 enculturated and 112 non-enculturated. Out of the 143 enculturated participants, 122 overlapped with a previous study [13]. The participants were asked to give demographic details before starting the survey. The participants residing in India who reported their native language as one of the languages specified in Indian constitution (Hindi, Bengali, Gujarati, Kannada, Malayalam, Tamil, Telugu, Marathi, Punjabi, Konkani, Sindhi, Oriya, and Urdu) were considered enculturated. The participants from the following countries, United States (18), UK (13), Hungary (62), France (3), Germany (3), Italy (2), Japan (2), Korea (1), Romania (1), Spain (2), and the Netherlands (3), not specified (2) were classified into the non-enculturated group. The demographic details of the participants are summarized in Table 1.

**Table 1:** Demographic details of participants.

Culture	N	Familiarity	N	Familiarity Score [Mean (SD)]
Enculturated	143 (56.08%)	Not at all	13 (9.09%)	2.86 (1.09)
		A Little	46 (32.17)	
		Somewhat	39 (27.27)	
		Very	34 (23.78)	
		Expert	9 (6.29)	
		Missing	2 (1.40)	
Non-Enculturated	112 (43.92%)	Not at all	48 (42.86%)	1.99 (1.02)
		A Little	26 (23.21)	
		Somewhat	29 (25.89)	
		Very	7 (6.25)	
		Expert	1 (0.89)	
		Missing	(0.89)	

### Characterization of the music stimuli

In order to understand the relative influences of two essential features of music, rhythm and tonality, in determining emotion ratings of enculturated and non-enculturated participants, quantitative estimates for rhythm and tonality were estimated as described below.

Rhythm in music is regarded as a complex temporal structure emerging from primary metrical structures such as the percept of a pulse (Pulse structures) and beat patterns (tempo) of the sample [27]. As a quantitative measure of the pulsation and tempo in the *raga* samples, pulse clarity and event density were used and were extracted using the MATLAB based Mir Toolbox v.1.5 [27] functions, *mirpulseclarity* and *mireventdensity*. The pulse clarity measures the clarity and stability of the beat in music and is estimated by the Shannon entropy of the fluctuation spectrum of a particular musical composition [28]. Music with easily perceived beats has a distinct and regular fluctuation spectrum and consequently has a high pulse clarity. The *mireventdensity* function measures the tempo by number of estimated note onsets per second for a musical excerpt.

*Gat* sections of the target *ragas* were characterized by significantly higher pulse clarity (0.43 ± 0.01), (0.04 ± 0.003), t (11) = -34.98, p < 0.001, and higher event density (1.47 ± 0.10) than the *alaap* sections (0.52 ± 0.03), t (11) = -10.63, p < 0.001 [13]. In other words, the *gat* stimuli were associated with greater rhythmic regularity and faster tempo than the *alaap* stimuli. This confirmed a clear distinction between *alaap* and *gat* in their rhythmic features. In order to avoid collinearity, and ensure simplicity in interpretation, rating variations between *alaap*/*gat* served as a binary proxy (0/1) for rhythmic variation in the regression models described below.

The second measure of interest was tonality, which is a central organizing principle in many different kinds of music wherein pitches are heard in relation to a tonic pitch [11]. An intrinsic characteristic feature of Hindustani music is the tonic drone, (usually played by a tanpura), which provides a reference to the listener, creating tonal relationships with the ‘solitary’ melody line. Since the drone is sounded throughout the presentation of the *raga*, any presentation mode of a *raga* piece can be viewed as a presentation of intervals, not just between notes of the melody line, but between each note and the *Sa* drone [17]. The tonality for each *raga* was thus obtained by taking the sum of the mean frequencies of occurrences of “minor” to “major” intervals. The major intervals are the *shuddh swaras* or the natural notes namely, the tonic, major second, major third, perfect fourth, perfect fifth, major sixth and major seventh while the minor intervals are the *komal swaras* (flat) positions of the same tones and are minor second, minor third, tritone, minor sixth and minor seventh.

The tonality of the *raga* was estimated by calculating frequency of occurrences of its corresponding tonic intervals. Tonic intervals are the difference in cents between the fundamental frequencies of the note being compared with the tonic and was determined using the method described by Bowling et al. (2012). First, the pitch was extracted for every 30 milliseconds window of the *raga* using the Melodia-Melody extraction toolbox [29] and represented in cents using Eq 1 below (where *f*_1_ is the frequency of the note in Hz and *f*_0_ is the frequency of the tonic in Hz),

$$\mathrm{Pitch}\;\left(\mathrm{cents}\right)=1200*\log_{2}\left(\frac{f_{1}}{f_{0}}\right)$$ (1)

Then, frequency of occurrence of each note/*swara* was estimated by collating the tonic intervals across each 100 cent bins spanning three octaves of a *raga* (labeled from −1200 to 2400 cents). Three octaves were then folded into one by adding the frequency of occurrence of the tonic intervals in each of the corresponding bins across the three octaves. For instance, the mean frequency of occurrence of minor second (*komal re*) would be the additive frequency of occurrence of the −1100, 100, and 1300 tonic interval cent bins. Finally, the ratio of the sum of mean frequencies of occurrences of the minor to the major intervals is calculated as the tonality of the *raga*. The tonalities for all the *ragas* used in this study were calculated for both presentation stages. As hypothesized no significant differences were seen in the tonality of the *ragas* across the two presentation modes. (S1 Fig, S1 Table).

### Data analysis

The emotional responses were analyzed using R statistical software (version 3.5.1) (https://www.r-project.org/). In accordance with the first hypothesis, two analyses were conducted wherein both groups were compared for emotional labels and intensity of ratings.

For all analyses, a mean data matrix of dimensions 24 (*raga* excerpts) X 8 (emotions) were created separately for the two cultures. Each matrix entry was an average of all participant ratings of the group for that particular excerpt and emotion, thus serving as an average of individual rating values.

#### Analysis for emotional responses–labels and intensity

Comparison of emotional labels–To compare the distributions of emotion labels by both cultural groups, two analyses were conducted. First a distribution of emotion labels for each raga excerpt was estimated, for both groups. The Kolmogorov-Smirnov test was used to test equality of distributions between the enculturated and non-enculturated groups. Next, the most frequently occurring emotion label for all the 24 (12 *ragas*, in 2 presentation stages) *raga* excerpts was calculated. The purpose of this analysis was to ascertain if the label of the highest rated emotion was similar between cultural groups. All two sided p-values less than 0.05 significance level were considered statistically significant.

Comparison of intensity rating–Following the comparison of labels, multiple two sample comparison of mean intensity ratings between the cultural groups was conducted by both Welch t-test (parametric) and Wilcoxon rank-sum test with continuity correction (non-parametric). In each case, 8 emotion ratings for 12 *ragas*, in 2 stages (*alaap*, *gat*) were compared, yielding a total of 192 (8×12×2) comparisons. The p-values from these 192 tests were corrected for multiple comparison error using Hochberg method. All the results and inferences were based on these adjusted p-values. As per these Null Hypothesis Significance Tests (NHST), “universality in emotion” perception was declared if more than 50% of adjusted p-values were greater than 0.05. Since the inability to reject the null does not imply equality of population means, the statistically non-significant cases were regarded as similar and therefore such cases were used as evidence towards universality.

#### Analysis to ascertain the role of tonality and rhythm

In order to investigate the role of rhythm and tonality in predicting the emotional responses, rigorous regression analyses were undertaken. After controlling for familiarity, rhythm (as a binary variable, *alaap* vs. *gat*) and tonality were used as predictors for emotional response. To increase confidence in the analyses and replicate the results, three separate methodologies were used to find the top predictors.

**Robust Linear Regression**

First, robust linear regressions were conducted to calculate proportion of variance explained by each predictor. Regression models were fitted using iterated reweighted least squares (IWLS) with bi-square psi function [30,31]as implemented in the “rlm” package in R (S2 Appendix).

**Odds Ratios**

Next, odds ratios were estimated based on proportions of variance extracted from the robust regression analyses. These odds ratios demonstrated the strength of association between musical features and the respective cultural groups and were calculated for each of the eight emotions using the following formula (E: Enculturated and NE: Non- Enculturated)

$$Odds\;Ratio=\frac{\%Variance\;\left(Tonality,E\right)/\%Variance\;\left(Rhythm,E\right)}{\%Variance\;\left(Tonality,NE\right)/\%Variance\;(Rhythm,NE)}$$ (2)

**Machine Learning**

All analyses conducted thus far, assumed linear model structure between emotional ratings and musical features. In order to validate this assumption without any implicit linearity, ML techniques were employed using Random Forest [32] and XGBoost [33]. Despite the fact that both ML techniques are ensemble tree-based methods, their innate model building processes fundamentally differ. Random Forest builds deep (full grown) parallel decision trees which are uncorrelated from each other [32], and thereby reduces the overall variance of the model. On the other hand, XGBoost is a scalable gradient boosting tree which builds weak (shallow) decision trees sequentially and optimizes based on an objective function [33] in order to reduce bias in the model. For this analysis, open source R packages of “randomForest” [34] and “xgboost” [33] were used.

For Random Forest, 500 trees with two predictors were grown and randomly sampled out of three at each split. In XGBoost, gradient tree booster was used with linear regression as the objective function. Root Mean Square Error (RMSE) was used for evaluation metric; other parameters were kept at their default for simplicity. As a measure of feature importance in Random Forest, the total decrease in Node Impurities from splitting on the feature, averaged over all trees was used and was measured by RMSE [34], the higher the value, the better predictive power of the feature. For XGBoost, “Gain” which ranks each predictor/feature based on relative contribution to the overall XGBoost model performance, was used as a measure of variable importance.

## Results

The mean familiarity score of enculturated participants was significantly higher than non-enculturated participants, t(245.09) = 6.49, p value < 0.00001 (without the assumption of homoscedasticity); thus justifying our cultural categorizations of enculturated and non-enculturated based on Indian/non-Indian nationality.

### Comparison of emotional response across enculturated and non-enculturated participants

**Fig 1:**
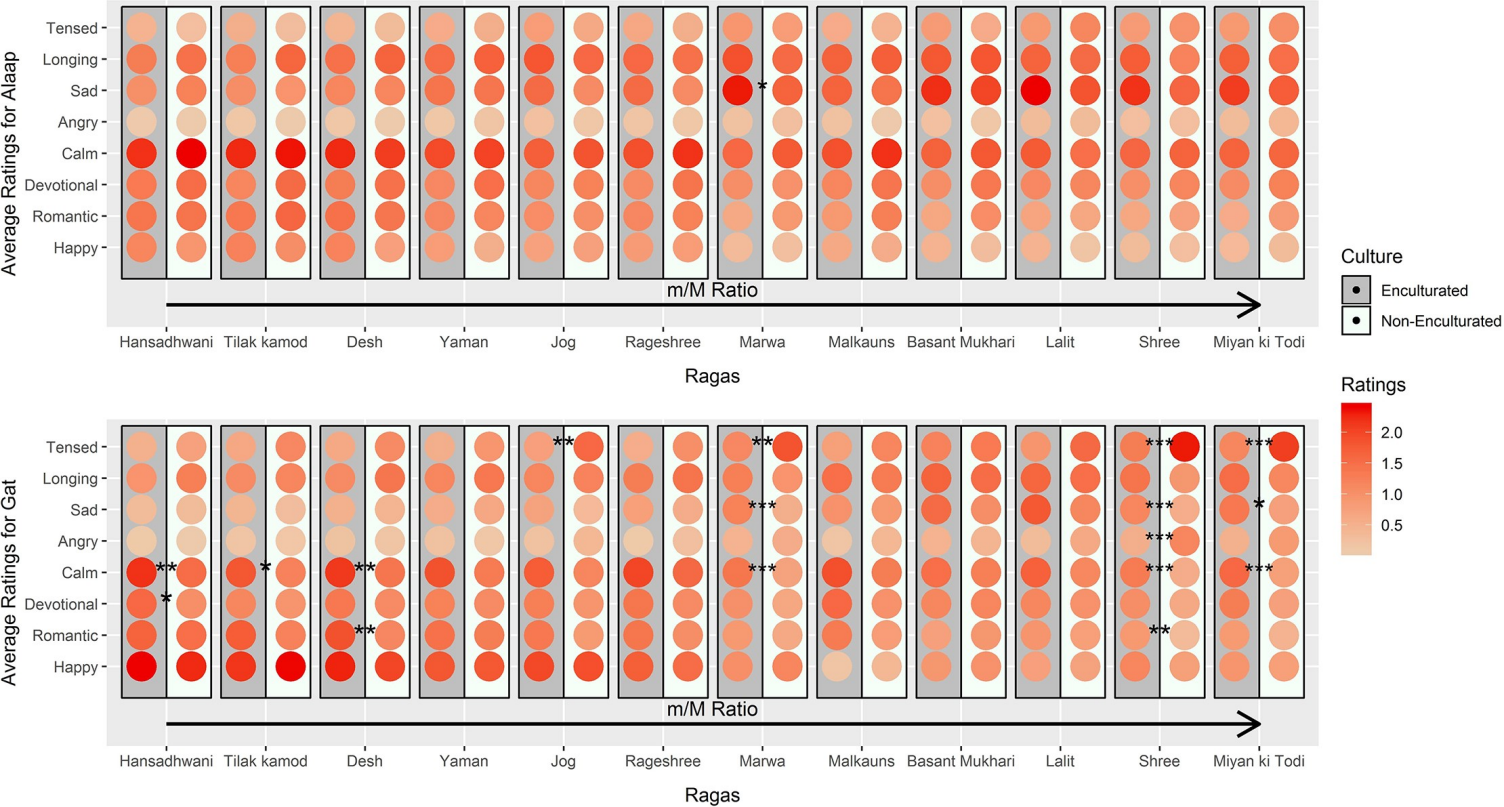
**Mean emotion rating comparisons, by Mode (Top: *Alaap*, Bottom: *Gat*), *Raga*, and Group (Enculturated/Non-Enculturated).** The different *ragas* (by name) are ordered on the x-axis while the y-axis represents the eight emotional labels. The grey background represents the ratings of enculturated group while the white background represents the rating of the non-enculturated group. The intensity of response is color coded as indicated by the color bar on the right. *Ragas* are ordered from lowest to highest tonal ratio (m/M) shows high degree of similarity in both label and intensity of emotion response across the cultural groups. Of the 192 comparisons, there were differences in just 18 cases as indicated in the figure. Adjusted p-values: *<0.05, ** < 0.01, *** < 0.001.

The mean emotional response ratings of enculturated (N = 143) and non-enculturated (N = 112) participants, for the *alaap* and *gat* of each *raga* are plotted in Fig 1. The alternating dark and light colored columns represent the emotion responses of the enculturated and non-enculturated groups respectively. The *ragas* are organized by their increasing tonal ratio (Number of minor intervals/Number of major intervals of the *raga)*. Earlier work has shown that as the tonal ratio increased, emotion ratings for *ragas* shift from positive to negative (plaintive) valence [35]. Visual comparison of the dark and light columns revealed similarity in label and intensity emotional responses between the enculturated and non-enculturated groups.

**Fig 2:**
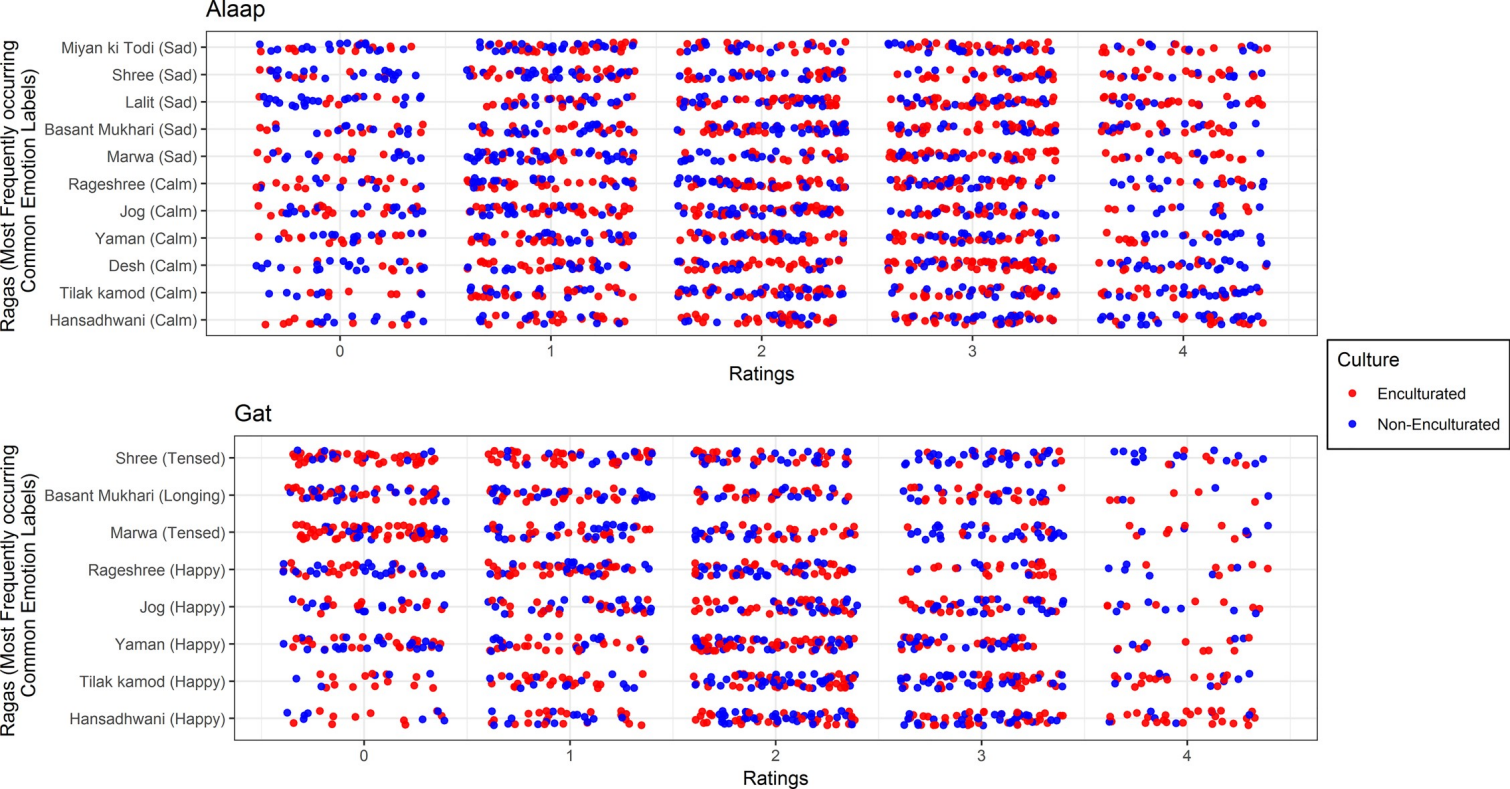
Dotplot of distribution of emotion ratings for most frequently occurring common emotions. A jittered dotplot of intensity ratings on x-axis of the most frequently occurring common emotion label associated with a raga on y-axis for the two cultural groups, *red for Enculturated and blue for Non-Enculturated) and two presentation stages. This dotplot avoids the problem of over-plotting due to discrete nature in this dataset by adding a small amount (horizontal variation of 40% and vertical variation of 20% of the resolution of the data) of random variation to the location of the ratings. The higher the concentration of dots by color, the higher the frequency of ratings by the cultural groups.

A comparison of the most frequently occurring emotion label for each of the 24 excerpts between the two cultural groups revealed similarities in 20 out of the 24 cases. A detailed dotplot describing the distribution of the ratings for those 20 excerpts by cultural groups is shown in Fig 2. The results of the Kolmogorov-Smirnov test in those 20 excerpts revealed significant differences in the distribution occurred only for Sadness ratings of *Marwa* and Tensed rating in *Gat* of both *Marwa* and *Shree* (S2 Table). Hence, sufficient evidence was not found to reject the null hypothesis of equality of distributions in more than 50% of the 24 excerpts.

Comparison of emotion rating intensities by both t-tests and Wilcoxon tests also revealed an equally high degree of agreement between the two cultural groups. In the t-tests, of the 192 comparisons, only 18 reached significant difference and in the Wilcoxon tests, only 19 emotion rating differences arose out of the 192 comparisons. These results indicate that over 90% of the emotion ratings were statistically indistinguishable between enculturated and non-enculturated participants. Of the significant differences that arose from the two tests, majority of them (17/18 in the t-test and 18/19 in the Wilcoxon test) were in the *gat* mode. The sole significant difference in *alaap* was observed in higher sadness ratings of *raga Marwa* by the enculturated group (p-value< 0.05). The results of the t-test comparisons are summarized in Fig 1, and a list of significant effects is provided in S3 Table. Among the 18 significant differences seen in the t-test comparisons, we decided to restrict our reporting to only those emotions with highest between participant group difference for a *raga* in a particular mode, which were 8 in number (S3 Table)

The analyses for distribution of most frequently occurring common emotion labels and the 192 simultaneous t-tests for equality of rating intensities overwhelmingly indicate universality in emotion ratings. Even though significant differences occurred in some comparisons, the proportion of such cases was very small. Since in both these analyses, more than 50% of the p-values were strictly greater than 0.05, we claim universality in emotion rating between the two cultural groups.

### Cultural differences in influences of rhythm and tonality on emotion ratings

#### Robust linear regression

To disentangle the relative influences of rhythm and tonality in emotional experience, and test whether these relative influences differ by cultural background, robust regression analysis was conducted. A total of 16 regressions (8 emotions versus. 2 groups) were conducted such that average emotion ratings of the twelve *ragas* were regressed with respect to rhythm and tonality after controlling for familiarity with Hindustani music.

**Fig 3:**
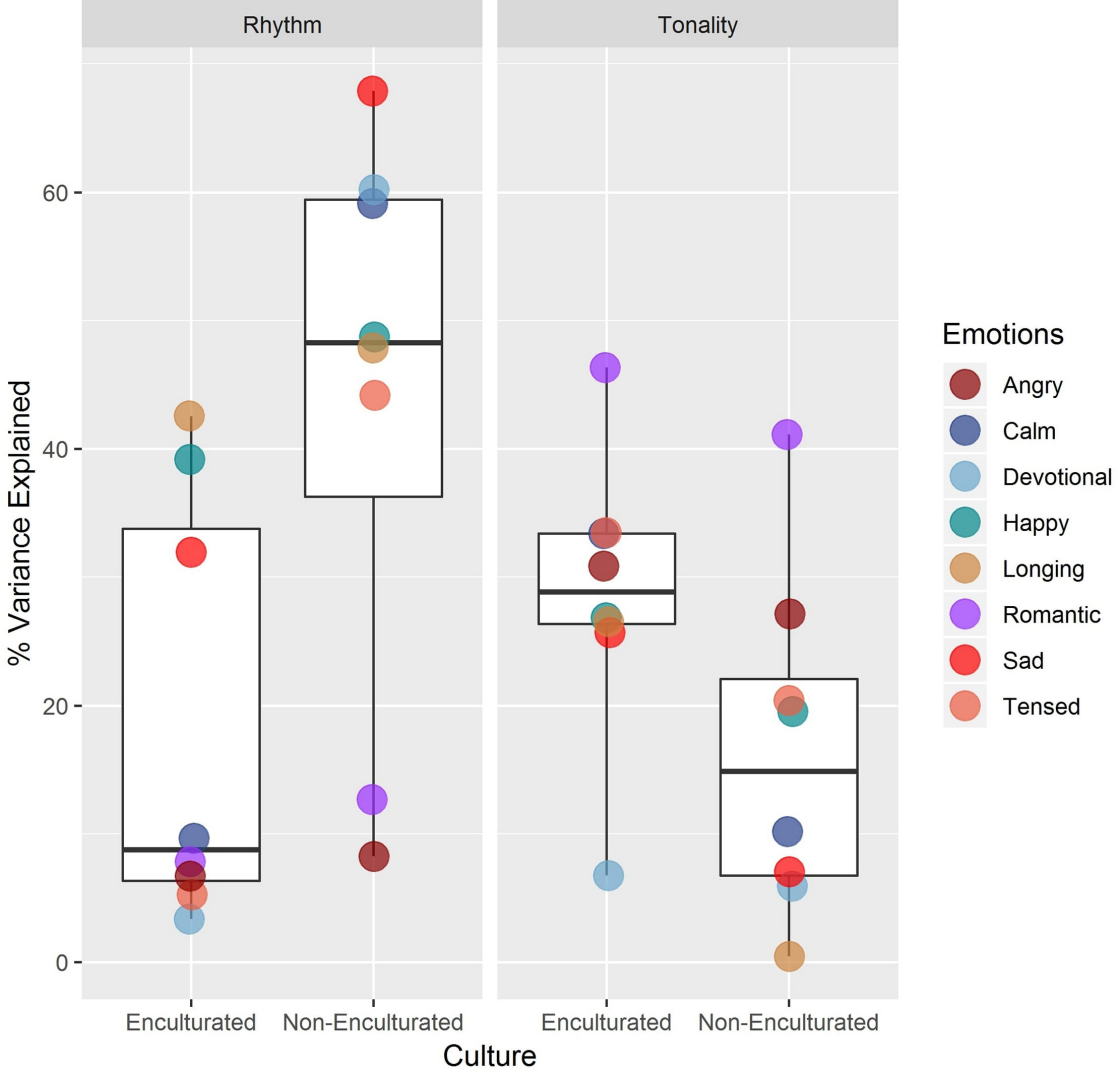
Box-and-whisker plots representing the percent variance explained in emotion ratings across two cultural groups. Median % variance explained is also displayed for each emotion individually.

The regression analysis revealed that the cues of tonality and rhythm regularity were used differentially by the enculturated and non-enculturated participants (Fig 3). As seen in Fig 3, the cue of tonality explained higher percent variance in emotion ratings for enculturated participants, compared to non-enculturated participants and thus emerged as a significant predictor for all emotions (except devotion) for the enculturated participants. On the other hand, for non-enculturated participants, rhythm explained a higher percent variance in emotions as compared to enculturated participants.

Familiarity with the stimuli was a partial contributor for some of the emotion ratings for both the enculturated and non-enculturated groups. Specifically, for enculturated participants, familiarity score explained the variance of emotion ratings in Happy (16%), Romantic (12%), Tensed (21%), Angry (19%) and Longing (6.4%). On the other hand, for non-enculturated participants, familiarity score explained the variance in Happy (16%) and Angry (17%). However, when compared to rhythm and tonality, familiarity played a much smaller role in the emotional response. The results are summarized in Table 2, and in Box and Whisker plots in Fig 3.

#### Odds ratios

The odds ratios for each group with musical features for each of the eight emotions are shown in Table 2. All the eight point estimates of odds ratios were greater than one and five of these were significant even after controlling for multiple comparison error. A common odds ratio was not calculated due to non-homogeneity between the odds ratios. Consistent with the results from robust regression, a significantly higher odds ratio for reliance on tonality was obtained for the enculturated group and for rhythm for the non-enculturated group.

**Table 2:** Percentage of variance explained by emotions and odds ratio.

Emotion		% Variance		F-value (4,20)		p-value		Odds Ratio with 95% CI	Adjusted p–value* for odds ratio
		E	NE	E	NE	E	NE		
**Calm**	*Tonality*	33.41	10.22	6.47	3.12	1.94 x 10^−2^	9.28 x 10^−2^	**19.95** (7.52, 52.94)	3.75 x 10^−11^
	*Rhythm*	9.69	59.15	0.52	15.95	0.48	7.13 x 10^−4^		
Happy*	*Tonality*	26.85	19.52	8.89	5.63	7.37 x 10^−3^	2.78 x 10^−2^	**1.71** (0.83, 3.51)	0.14
	*Rhythm*	39.17	48.71	61.12	74.64	1.66 x 10^−7^	3.48 x 10^−8^		
**Sad**	*Tonality*	25.69	7.03	6.36	2.13	2.0 x 10^−2^	0.16	**7.76** (3.05, 19.75)	3.51 x 10^−6^
	*Rhythm*	31.98	67.90	21.72	44.55	1.5 x 10^−4^	1.70 x 10^−6^		
**Tensed**	*Tonality*	33.48	20.40	5.44	6.73	3.0 x 10^−2^	1.7 x 10^−2^	**13.78** (4.78, 39.71)	7.67 x 10^−8^
	*Rhythm*	5.26	44.17	0.19	9.98	0.67	4.9 x 10^−3^		
**Longing**	*Tonality*	26.57	0.48	8.15	0.07	9.78 x 10^−3^	0.80	**62.26** (3.48, 1113.73)	2.32 x 10^−6^
	*Rhythm*	42.55	47.86	34.69	9.00	9.24 x 10^−6^	7.10 x 10^−3^		
**Angry**	*Tonality*	30.88	27.16	11.26	32.74	3.15 x 10^−3^	1.34 x 10^−5^	**1.40** (0.45, 4.38)	0.56
	*Rhythm*	6.71	8.26	0.02	0.59	0.88	0.45		
**Devotional**	*Tonality*	6.76	5.95	0.68	2.08	0.42	0.16	**20.24** (4.28, 95.73)	5.12 x 10^−6^
	*Rhythm*	3.38	60.22	3.10	18.92	0.09	3.11 x 10^−4^		
**Romantic**	*Tonality*	46.34	41.12	16.72	8.92	5.72 x 10^−4^	7.30 x 10^−3^	**1.82** (0.68, 4.88)	0.23
	*Rhythm*	7.84	12.68	12.46	0.70	2.10 x 10^−3^	0.41		

Percentages of Emotion Variance Explained, F-values, p-values of regression effect size, Odds Ratios and confidence intervals (CI) for Tonality vs. Rhythm; by Emotion, and Cultural Group (E = Enculturated, NE = Non-Enculturated), across all ragas.

* Hochberg Method

#### Machine learning

**Fig 4:**
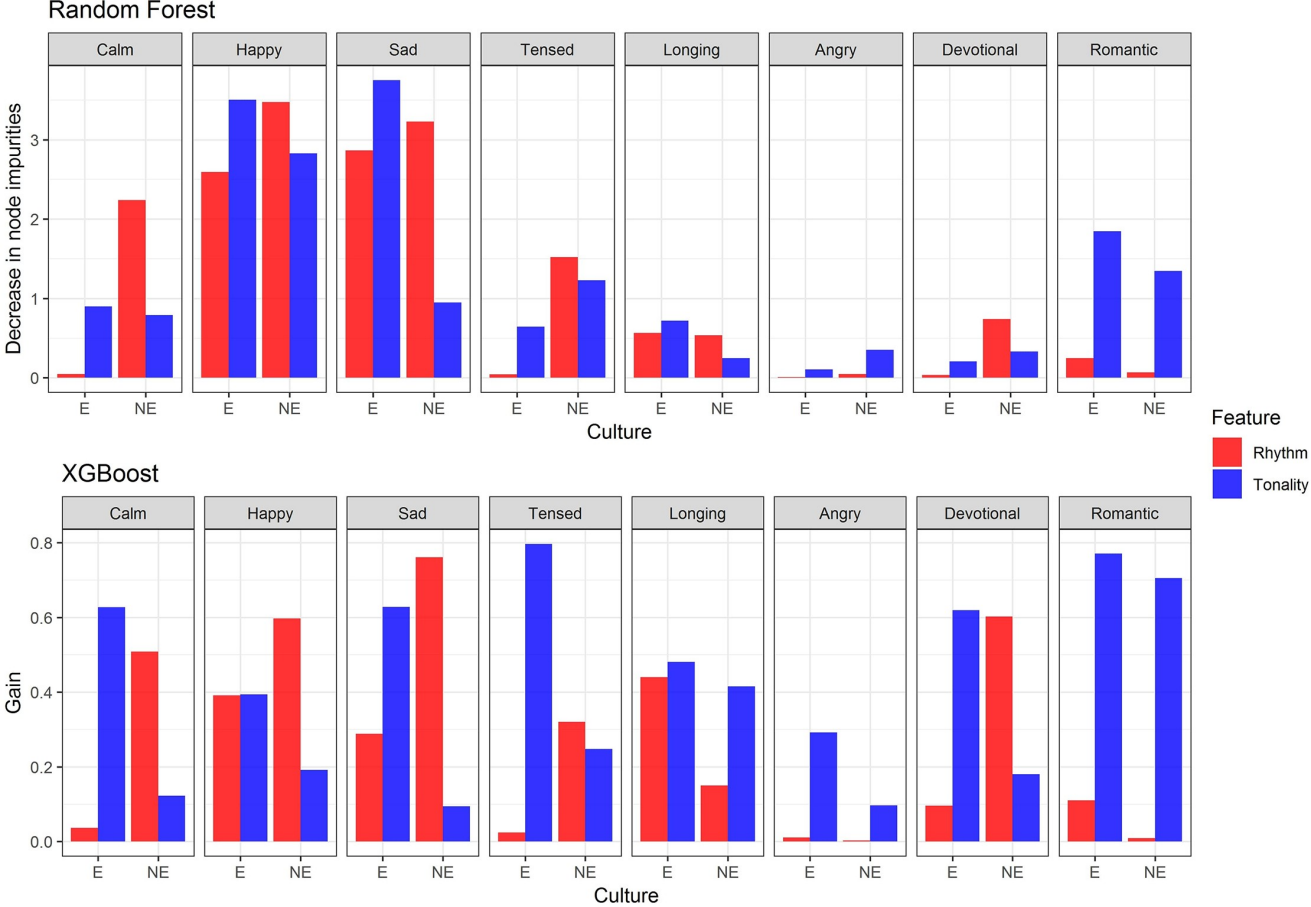
Bar plot of Variable Importance measures. Based on Random Forest and XGBoost, Variable Importance of Musical Features (Rhythm and Tonality) are plotted for modeling emotional ratings for each of the eight emotions and two cultural groups (E = Enculturated, NE = Non-Enculturated).

The Machine Learning (ML) analysis was conducted similar to the robust regression setup. A total of 16 models (8 emotions x 2 groups) were evaluated for each of the methods with average emotion ratings of the twelve *ragas* as the response and tonality, rhythm, familiarity with Hindustani music as predictive features. The results are presented in S4 Table and plotted in Fig 4.

The results from both the ML methods were very similar and confirm our earlier inference from linear robust regressions (Fig 4, S4 Table) and odds ratio. For the enculturated group, tonality was always a better predictive feature than rhythm for all emotions in both the methods. For non-enculturated group, rhythm was a better predictive feature than Tonality for all the emotions except for Angry and Romantic in Random Forest and Angry, Romantic and Longing in XGBoost.

## Discussion

We compared for the first time, emotion responses for different Hindustani *ragas*, across both *alaap* and *gat* for two cultural groups, one familiar and the other unfamiliar with *raga* music. Our results indicate that (1) the emotions experienced by individuals with little to no exposure to Hindustani *raga* music (non-Indians/non-enculturated) are highly similar to those reported by individuals enculturated to *raga* music (Indians/enculturated), and (2) For Indian participants, the tonality of *ragas* emerged as a larger determinant of emotion ratings than rhythm, whereas for non-Indians, rhythm was the more determinant of emotion ratings.

Emotional responses across the two groups were compared for both label and intensity. As indicated in Fig 1, of the 192 comparisons of enculturated and non-enculturated listeners’ ratings of 8 different emotions and both *alaap* and *gat* versions of 12 different *ragas*, more than 50% of the p-values were strictly greater than 0.05 and significant differences arose in only 18 cases. Of these 18 cases, only 4 were attributed to differences in label (Fig 2) and the remaining were due to differences in intensity. In other words, Indian and Non-Indian participants listening to the same *ragas* reported different emotional experiences less than 10% of the time. Thus, our results show similarity across cultures for both positive and negative musical emotions and provide new compelling evidence for universality in the auditory domain in the realm of musical emotions. In addition, we report universality across cultures for emotions hitherto not reported earlier. Past emotion recognition studies have suggested that anger, disgust, fear, happiness, sadness, and surprise are universally recognized [36–40]. We add positive emotions like romantic, calm and longing to this list. Since our analysis investigated both label and intensity of emotional response, we also show for the first time, similarity in the naming of emotional labels across cultures. Our questionnaire used bilingual labels, namely those used within Indian culture along with the closest western translations (counterparts). We therefore suggest that studies that compare emotional responses across cultures might consider adopting similar aspects to enable fair cultural comparisons of emotion labels.

Of the 18 differences in emotion intensity ratings, (out of 192), only one difference arose for *alaap* (Sad ratings for *raga Marwa*), suggesting that the temporal rigidity that is imposed on the *raga* scales during the *gat* rendition either caused, or revealed underlying cultural differences in the way our listeners experienced the same musical stimuli. Most of the differences that did arise, however, were confined to certain *ragas*, and certain emotions (see Fig 1). Of the 17 significant differences in *gat* emotion ratings, for instance, 5 were for emotions experienced while hearing the *gat* of *raga Shree*, three were for *raga Marwa*, and three were for *raga Miyan ki Todi*; 9 of these differences were for Tensed, Sad, and Calm ratings for these *ragas*. These findings also reiterate that distinct emotion labels elicited by Hindustani *ragas* are evident more prominently in the *gat* mode rather than the *alaap*. Taken together with the similarity in the distribution of ratings for the most frequently occurring common emotion labels, evidence decidedly favors a shared emotional experience among Indian and non-Indian listeners. This is in agreement with findings reported for Hindu classical dance [41] wherein participants from the American culture were found to be quite accurate and similar to those from India in identifying emotion.

In lieu of the finding that the emotions reported by the two cultural groups were largely indistinguishable, statistically speaking, our second objective, testing the relative influences of tonal and rhythmic cues on each group’s emotion ratings, assumed new significance, since a lack of group differences at this stage would heavily favor musical universality.

The results of our robust regression analyses revealed an interesting cross-cultural difference in the degree to which rhythm and tonality influenced the musical emotions of our participants as they listened to each *raga*. For the Indian (enculturated) sample, variations in the *tonality* of different *ragas* determined emotion ratings more so than did rhythmic cues (Fig 3, Table 2). For the non-Indian (non-enculturated) sample, rhythm influenced ratings more than tonality. Tonality was a significant predictor of 7 out of 8 emotions for the Indian group, but only 5 of 8 emotions in the non-Indian group. Rhythm, meanwhile, predicted only 4 of the 8 emotions in the Indian group, but in non-Indians it predicted 6 out of 8 emotions. The emotion rating variances explained by tonality versus rhythm in each group offered further support for this cross-cultural difference; this greater influence of tonality in enculturated participants, and rhythm in the non-enculturated group, is best encapsulated in the box plots in Fig 3. These results were strengthened by the odds ratio calculated for percent variance explained by the regression analysis (Table 2). Machine learning methods Random Forest and XGBoost further confirmed the results obtained in linear regressions (Fig 4, S4 Table). Most importantly, we converged to the same result using “percent variance explained” in robust regression, “Decrease in node impurity” in Random Forest and “Gain” in XGBoost.

In effect, our analyses suggest enculturated and non-enculturated participants reported universal musical emotions listening to our *raga* stimuli, but they converged on their emotional experiences via two different cues, tonality and rhythm, respectively. In terms of the universality-vs.-culture debate, these results offer a third possibility: universality in musical emotion, but via different musical cues to emotion depending on listeners’ cultural backgrounds. In terms of the CRM model, since the enculturated participants relied upon tonality as the primary cue when rating their emotions, we suggest ‘tonality’ as a key component of Balkwill and Thompson’s [7] second “lens”. That our non-enculturated sample relied upon rhythmic cues in light of tonal unfamiliarity is consistent with the proposal that the use of an isochronous (equally timed) beat as a universal feature [9] and recent work showing that a rhythmic sense in music may be more inherent, and universal, than tonal sense [42]. Similar results have been reports in cultural groups matched for tonal familiarity, but which differ in formal music training, with training in music accentuating the reliance on tonality cues more than the rhythmic cues [43]. Likewise, just as Castellano et al., [35] found that Indian listeners had a greater sensitivity to the uniquely Indian tonal hierarchies underlying Hindustani *ragas*, compared to American university students, the results of our regression and machine learning analyses suggest that tonality was a particularly salient emotional cue for our Indian participants, whereas our non-Indian comparison group depended more on rhythmic variation to glean emotion from our *raga* stimuli.

Finally, a notable feature of this study which distinguishes it from others in the debate over the universal musical emotions is its use of *ragas*. Whilst these compositions have a history millennia old, and are an integral part of the rich culture of North India, the present study demonstrates well their utility as experimental stimuli, and their potential value in future work. The canon of *Hindustani ragas* is a ready-made palette for musical emotion studies requiring graded degrees of emotional valence which also allows a variety of positive and negative emotions in music to be investigated. While the nature of the music survey, which was conducted online, restricted our control over the use of varied hearing devices by the participants, nevertheless, we hope this study not only opens a new window into an expanded taxonomy of musical emotions but also highlights the use of acoustic features to characterize auditory stimuli and thereby provide a more comprehensive understanding of emotions in the auditory domain.

## Supporting information

S1 Appendix Comparison of tonality across *alaap* and *gat* of the *ragas*. (PDF)

S2 Appendix Model fitting in robust regression. (PDF)

S1 Fig Stacked bar plots of mean frequency of occurrences. Stacked Bar plots representing Mean frequency of Occurrences of each 12 Notes across *Alaap* and *Gat* for all the 12 *Ragas*.
(TIFF)

S1 Table Tonality for all the *Raga*. (PDF)

S2 Table Table for comparing the distributions of ratings for most frequently occurring common emotion labels. Comparison of distribution of ratings by cultural groups based on the common most frequently occurring emotion label.
(PDF)

S3 Table Table for highest significant differences. Highest significant differences for each raga in a particular mode arising from enculturated versus non-enculturated t-test comparisons of emotion ratings (E = Enculturated, NE = Non-Enculturated).
(PDF)

S4 Table Table for Variable Importance Measures. Variable Importance measure (based on Random Forest and XGBoost) of Musical Features (Rhythm and Tonality) for modeling emotional ratings for each of the eight emotions and Enculturation Group (E = Enculturated, NE = Non-Enculturated).
(PDF)
