# Supplementary material for: Cultural differences in the use of acoustic cues for musical emotion experience
Source: PLoS One. 2019 Sep 13;14(9):e0222380. doi: 10.1371/journal.pone.0222380 (PMC6743780; doi:10.1371/journal.pone.0222380)
Supplement: S1 Appendix — (PDF) [file pone.0222380.s001.pdf]

S1 Appendix. Comparison of tonality across *alaap* and *gat* of the *ragas*.

In order to verify that tonality across the two modes of rendition does not differ significantly, the following analyses were conducted. The mean frequency of occurrences of each of the 12 notes across *Alaap* and *Gat* for all the 12 *Ragas* is calculated as described in the methods section and is shown in S1 Fig. A paired t-test (parametric) was used to compare the mean frequency of occurrences of notes between *Alaap* and *Gat* for each of the 12 *ragas* (S1 Table). This was followed by Wilcoxon test, where the median frequency of occurrence was compared between the two modes of each *raga*. Additionally, a Kolmogorov Smirnov test was conducted, to check if the distributions of frequencies for the two modes differed significantly. Separately in all the three tests, the p-values are greater than 0.05, and ranged from (0.99 - 1) and (0.68 - 1) and (0.85 - 0.99) for paired t-test, Wilcox signed rank test and Kolmogorov Smirnov test respectively. This confirmed that the tonality of a *raga* across the two presentation stages is similar.
