# Supplementary material for: Cultural differences in the use of acoustic cues for musical emotion experience
Source: PLoS One. 2019 Sep 13;14(9):e0222380. doi: 10.1371/journal.pone.0222380 (PMC6743780; doi:10.1371/journal.pone.0222380)
Supplement: S2 Appendix — (PDF) [file pone.0222380.s002.pdf]

S2 Appendix. Model fitting in Robust Regression.

Parameters were estimated using M-estimation and for scale estimation, re-scaled MAD (Median Absolute Deviation) of the residuals was used. “M-estimation” is an estimation technique introduced by Huber for the robust regressions. “MAD” (Median Absolute Deviation) is a robust measure of the variability used to estimate the scale parameter in the regressions. Since in robust regression there are multiple ways to estimate parameters, we have specified the particular methods undertaken by us as implemented in R. This would facilitate future replications.
