# Supplementary figures and images for: Cultural differences in the use of acoustic cues for musical emotion experience
Source: PLoS One. 2019 Sep 13;14(9):e0222380. doi: 10.1371/journal.pone.0222380 (PMC6743780; doi:10.1371/journal.pone.0222380)

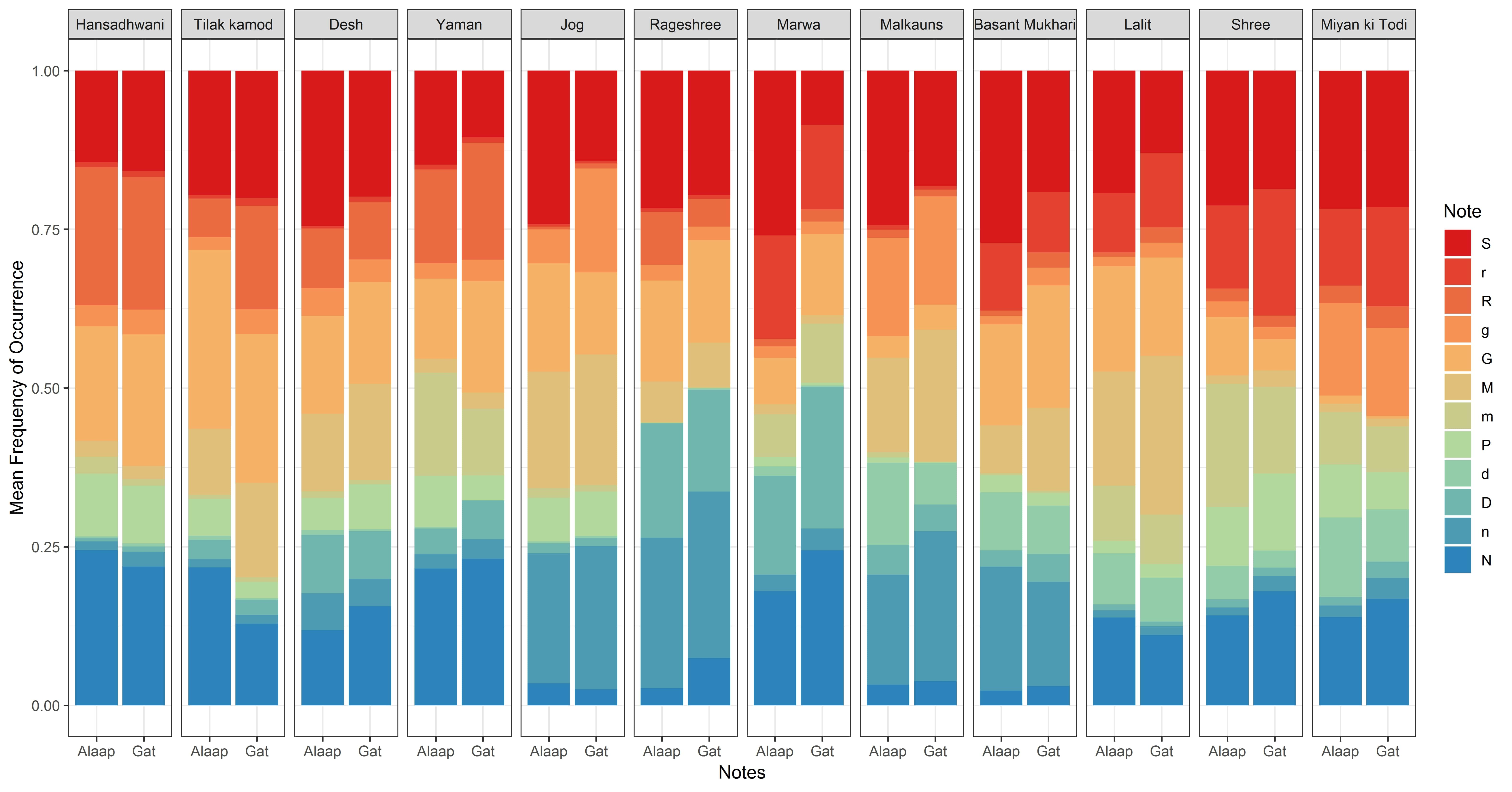

Supplement: S1 Fig — Stacked Bar plots representing Mean frequency of Occurrences of each 12 Notes across Alaap and Gat for all the 12 Ragas. (TIFF) [file pone.0222380.s003.tiff]
