# Supplementary material for: Cultural differences in the use of acoustic cues for musical emotion experience
Source: PLoS One. 2019 Sep 13;14(9):e0222380. doi: 10.1371/journal.pone.0222380 (PMC6743780; doi:10.1371/journal.pone.0222380)
Supplement: S1 Table — (PDF) [file pone.0222380.s004.pdf]

S1 Table. Tonality for all the *Raga*

Raga	**Tonality (*Alaap*)**	**Tonality (*Gat*)**
Hansadhwani	0.091	0.095
Tilak kamod	0.054	0.082
Desh	0.140	0.107
Yaman	0.281	0.217
Jog	0.389	0.684
Rageshree	0.368	0.414
Marwa	0.407	0.395
Malkauns	0.897	0.920
Basant Mukhari	0.694	0.579
Lalit	0.402	0.433
Shree	0.708	0.682
Miyan ki Todi	0.966	0.932
