# Supplementary material for: Cultural differences in the use of acoustic cues for musical emotion experience
Source: PLoS One. 2019 Sep 13;14(9):e0222380. doi: 10.1371/journal.pone.0222380 (PMC6743780; doi:10.1371/journal.pone.0222380)
Supplement: S2 Table — Comparison of distribution of ratings by cultural groups based on the common most frequently occurring emotion label. (PDF) [file pone.0222380.s005.pdf]

S2 Table. Table for comparing the distributions of ratings for most frequently occurring common emotion labels. Comparison of distribution of ratings by cultural groups based on the common most frequently occurring emotion label; KS: Kolmogorov Smirnov Test; p-values were adjusted by Hochberg Method

Mode	Raga	Most Frequently Occurring Common Emotion Lables	KS (Raw p-value)	KS (Adjusted p-value)
Alaap	Hansadhwani	Calm	0.322	1.000
Alaap	Tilak kamod	Calm	0.984	1.000
Alaap	Desh	Calm	0.265	1.000
Alaap	Yaman	Calm	0.987	1.000
Alaap	Jog	Calm	0.948	1.000
Alaap	Rageshree	Calm	0.893	1.000
Alaap	Marwa	Sad	0.001	0.015
Alaap	Basant Mukhari	Sad	0.905	1.000
Alaap	Lalit	Sad	0.091	1.000
Alaap	Shree	Sad	0.056	0.898
Alaap	Miyan ki Todi	Sad	0.526	1.000
Gat	Hansadhwani	Happy	0.294	1.000
Gat	Tilak kamod	Happy	0.468	1.000
Gat	Yaman	Happy	1.000	1.000
Gat	Jog	Happy	0.955	1.000
Gat	Rageshree	Happy	0.970	1.000
Gat	Marwa	Tensed	0.001	0.005
Gat	Basant Mukhari	Longing	1.000	1.000
Gat	Shree	Tensed	0.000	0.000
