# Supplementary material for: Cultural differences in the use of acoustic cues for musical emotion experience
Source: PLoS One. 2019 Sep 13;14(9):e0222380. doi: 10.1371/journal.pone.0222380 (PMC6743780; doi:10.1371/journal.pone.0222380)
Supplement: S3 Table — Highest significant differences for each raga in a particular mode arising from enculturated versus non-enculturated t-test comparisons of emotion ratings (E = Enculturated, NE = Non-Enculturated). (PDF) [file pone.0222380.s006.pdf]

S3 Table. Table for Highest significant differences. Highest significant differences for each raga in a particular mode arising from enculturated versus non-enculturated t-test comparisons of emotion ratings (E=Enculturated, NE=Non-Enculturated)

Raga	***Alaap /Gat***	**Emotion**	**Average Rating by E**	**Average Rating by NE**	**t value**	**Raw p-value**	**Adjusted p-value**
Marwa	Alaap	Sad	2.34	1.67	3.89	1.34×10^{-4}	< .05
Hansadhwani	Gat	Calm	2.12	1.47	4.28	2.92×10^{-5}	< .01
Tilak kamod	Gat	Calm	1.77	1.17	3.83	1.70×10^{-4}	< .05
Desh	Gat	Calm	2.07	1.33	4.35	2.33×10^{-5}	< .01
Jog	Gat	Tensed	0.75	1.53	-4.56	9.37×10^{-5}	< .01
Marwa	Gat	Sad	1.20	0.51	5.05	9.56×10^{-7}	< .001
Miyan ki Todi	Gat	Calm	1.54	0.74	5.28	3.22×10^{-7}	< .001
Shree	Gat	Tensed	1.26	2.26	-5.95	1.21×10^{-8}	< .001
