# Supplementary material for: Cultural differences in the use of acoustic cues for musical emotion experience
Source: PLoS One. 2019 Sep 13;14(9):e0222380. doi: 10.1371/journal.pone.0222380 (PMC6743780; doi:10.1371/journal.pone.0222380)
Supplement: S4 Table — Variable Importance measure (based on Random Forest and XGBoost) of Musical Features (Rhythm and Tonality) for modeling emotional ratings for each of the eight emotions and Enculturation Group (E = Enculturated, NE = Non-Enculturated). (PDF) [file pone.0222380.s007.pdf]

S4 Table. Table for Variable Importance measures. Variable Importance measure (based on Random Forest and XGBoost) of Musical Features (Rhythm and Tonality) for modeling emotional ratings for each of the eight emotions and Enculturation Group (E=Enculturated, NE=Non-Enculturated).

**Emotion**		**Total Decrease in node impurities (Random Forest)**		**Gain (XGBoost)**	
		**E**	**NE**	**E**	**NE**
**Calm**	*Tonality*	0.897	0.791	0.627	0.122
	Rhythm	0.048	2.239	0.037	0.509
**Happy**	*Tonality*	3.506	2.827	0.394	0.192
	Rhythm	2.595	3.476	0.391	0.597
**Sad**	*Tonality*	3.749	0.951	0.628	0.095
	Rhythm	2.865	3.227	0.289	0.761
**Tensed**	*Tonality*	0.645	1.227	0.796	0.247
	Rhythm	0.045	1.520	0.024	0.320
**Longing**	*Tonality*	0.719	0.246	0.481	0.416
	Rhythm	0.564	0.537	0.440	0.150
**Angry**	*Tonality*	0.108	0.352	0.292	0.097
	Rhythm	0.009	0.046	0.011	0.003
**Devotional**	*Tonality*	0.207	0.330	0.619	0.180
	Rhythm	0.035	0.741	0.096	0.603
**Romantic**	*Tonality*	1.846	1.347	0.771	0.705
	Rhythm	0.249	0.069	0.110	0.009
